# Effect of misoprostol with and without evening primrose (*Oenothera biennis*) on induction of missed abortion

**DOI:** 10.22038/AJP.2023.22179

**Published:** 2023

**Authors:** Mozhgan Mahmoodinasab, Marzeyeh Loripoor, Reza Vazirinejad, Fariba Aminzadeh

**Affiliations:** 1 *Department of Midwifery, School of Nursing and Midwifery, Rafsanjan University of Medical Sciences, Rafsanjan, Iran*; 2 *Department of Midwifery, School of Nursing and Midwifery; Geriatric Care Research Center, Rafsanjan University of Medical Sciences, Rafsanjan, Iran*; 3 *Department of Epidemiology and Statistics, School of Medicine, Social Determinants of Health Research Center, Rafsanjan University of Medical Sciences, Rafsanjan, Iran*; 4 *Department of Obstetrics and Gynecology, Faculty of Medicine, Nik Nafs Educational and Medical Center, Rafsanjan University of Medical Sciences, Rafsanjan, Iran*

**Keywords:** Missed abortion, Misoprostol, Evening primrose, Cervix preparation

## Abstract

**Objective:**

To determine whether addition of evening primrose to a misoprostol-based abortion regimen can increase the success of abortion.

**Materials and Methods:**

In this randomized clinical trial., 148 women referring to Niknafas Hospital in Rafsanajn with diagnosis of missed abortion were randomly allocated into two 74-subject groups. The intervention group used 2000 mg vaginal evening primrose capsules the night before the hospitalization, while the control group did not receive any medication. Both groups received an initial dose of 800 μg of vaginal misoprostol after admission and the next dose was given three hours later if necessary.

**Results:**

The two groups had significant differences in terms of full abortion, consistency and dilatation of cervix, duration between the first dose of misoprostol until the ejection of fetus, the misoprostol dose administered, and the level of vaginal bleeding during the hospitalization. They had no significant differences regarding curettage, duration of hospitalization, or side effects. The mean pain score had no significant difference between the two groups, though the score was lower in the intervention group (p>0.05).

**Conclusion:**

Administration of vaginal evening primrose before vaginal misoprostol was found to be more effective compared to misoprostol alone in missed abortion.

## Introduction

Missed abortion is a common and constantly growing condition observed in 15-20% of diagnosed pregnancies (Neilson et al., 2013 ▶; Jurkovic et al., 2013 ▶, Graziosi et al., 2004 ▶; Muffley et al., 2002 ▶; Niinimäki et al., 2011 ▶) and it has been defined as empty pregnancy sac or fetus without cardiac activity and without spontaneous abortion (Neilson et al., 2013 ▶).

Treatment of missed abortion includes one of the anticipation, pharmacological, and surgical methods (Jurkovic et al., 2013 ▶). The surgical method of discharging the uterus contents has long been the standard treatment (Graziosi et al., 2004 ▶). Nevertheless, it has the risks of damaging the uterus and cervix, uterine adhesions, bleeding, infection, possible anesthesia complications, and possible adverse effects on subsequent pregnancies (Niinimäki et al., 2011 ▶; Graziosi et al., 2004 ▶). The results of a meta-analysis on comparing the anticipation and surgical methods indicated that the risk of infection and psychological problems was similar in both methods, but the costs are lower in the anticipation method. Nevertheless, in the anticipation method, incomplete abortion and the need for surgery to discharge the uterus contents, increased bleeding, and blood transfusion are more probable (Nanda et al., 2012 ▶).

Pharmacotherapy is the preference of most women, while also being recommended by international clinical guidelines (Chen and Creinin, 2007 ▶; Zhang et al., 2005 ▶). The success rate of medical treatment is very variable and has been reported to range from 13 to 90% (Gemzell‐Danielsson et al., 2007 ▶). Misoprostol is commonly used for medical treatment (Robledo et al., 2007 ▶). According to the International Federation of gynecology and obstetrics in 2017, the recommended dose of misoprostol for treating missed abortion is 800 μg every three hours up to two doses vaginally, or 600 μg every three hours up to two doses sublingually (Gemzell‐Danielsson, 2007 ▶). Use of misoprostol is not always successful as 15-40% of cases need additional misoprostol doses which leads to prolongation of the treatment process (Obstetrics, 2017 ▶; Neilson et al., 2006 ▶; Chen and Creinin, 2007 ▶; Zhang et al., 2005 ▶; Robledo et al., 2007 ▶; Creinin et al., 2006 ▶).

Use of mifepristone before misoprostol boosts the abortion-inducing properties of misoprostol, causing complete abortion in 95% of pregnancies with the age of 65 days after fertility and 93% of pregnancies with 64-70 days (Chen and Creinin, 2015 ▶; Abbas et al., 2015 ▶). Although the national Institute for health and care excellence (NICE) did not show any benefit of applying mifepristone before misoprostol based on the results of a clinical trial in 2012 and thus did not recommend administering mifepristone before misoprostol, later the results of a large double-blind clinical trial controlled with placebo indicated that the combination of mifepristone and misoprostol would have a better impact on discharging the uterus (Chu et al., 2020 ▶). Nevertheless, mifepristone is expensive and unavailable in many centers (Raymond et al., 2017 ▶). Accordingly, it seems identification of an effective and yet less expensive and more available drug would be practical and useful to boost the effect of misoprostol in cases of missed abortion.

The use of herbal medicine as an alternative treatment has been embraced throughout the world (Dermani, 2021 ▶). The oil of evening primrose (*Oenothera biennis*) contains large amounts of linoleic acid and gamma-linoleic acid, which directly converts unsaturated fatty acid to prostaglandin (Babazadeh and Keramat, 2011 ▶). Various studies have shown its impact on cervix preparation before or during induction of labor as well as before gynecology operations that require cervix preparation (Najafi et al., 2019 ▶; Shahali et al., 2018 ▶; Vahdat et al., 2015 ▶).

Considering the effects observed for evening primrose in studies and since so far no research has been conducted regarding the effect of the plant alongside misoprostol in treating missed abortion, this study was performed to investigate the effect of misoprostol with and without evening primrose on the induction of missed abortion 

## Materials and Methods

This randomized clinical trial was performed on women diagnosed with missed abortion referring to Niknafas maternity Hospital in Rafsanjan from February 2020 to June 2020, as approved by the ethics committee of Rafsanajn University of medical sciences with the ethics code of IR.RUMS.REC.1398.146 and code IRCT20160308026971N8 from Iranian registry of clinical trials.

 The sample size was calculated as 59 in group based on the study by Jamilian et al. (Jamilian et al., 2015 ▶), with confidence interval of 95% and statistical power of 80%. Considering possible attrition, 74 subjects were considered in each group. The inclusion criteria were diagnosis of missed abortion based on two sonography tests (as confirmed by the second sonography or with a 7-day interval by the same sonographer), signing written informed consent form to participate in the study, no contraindication use of evening primrose and misoprostol, and no severe vaginal bleeding. In case of not using evening primrose or improper using as well as, start of bleeding before using evening primrose and referral to the surrounding cities for treatment follow-up, the subject would be excluded.

For random allocation of the subjects into the intervention and control groups, the simple randomization method was used, number 1 was considered for intervention and 2 for the control group, and cards 1 and 2 were placed inside in a box in which the numbers were not visible. The first person would pick up one card and enter the relevant group. The next person would be allocated to the opposite group without picking up any cards, until the samples were completed. Both groups were recommended to refer in the morning of the next day for induction of abortion, with the intervention group used two 1000-mg evening primrose capsules vaginally made by Barij pharmaceutical company at night before sleep, while the control group did not receive any intervention. Similar studies were used to determine the dose of evening primrose (Nonette, 2017 ▶; Vahdat et al., 2015 ▶; Kalati et al., 2018 ▶; Girlie, 2015 ▶). In the next morning, once the participants referred to the study center, after examining vital signs, bleeding, and cervix status, their hospitalization file was created and 800 μg of misoprostol suppository made by Samisaz pharmaceutical company (Mashhad, Iran) was embedded in the posterior fornix for both groups. The next dose would be repeated three hours later if necessary. The next morning abdominal sonography was performed to diagnose complete or incomplete abortion for all participants.

The data related to the studied variables was extracted from the women's files, while the information including pain score, nausea, diarrhea, headache, abdominal cramps, fever and chills that were not recorded in the file was asked by a trained expert and recorded in a checklist. To determine the pain intensity, visual analog scale (VAS) was used and scores 1-3 represented mild, 4-7 moderate, and 8-10 severe pain (Bikmoradi et al., 2014 ▶).

The collected data were inputted into SPSS 16 and chi-square test was used for qualitative variables while independent t-test was employed for comparing quantitative variables of the two groups. In some cases, its alternative test, i.e. Fisher exact test was used to compare the two groups. A p<0.05 was considered statistically significant.

## Results

**Figure 1:**
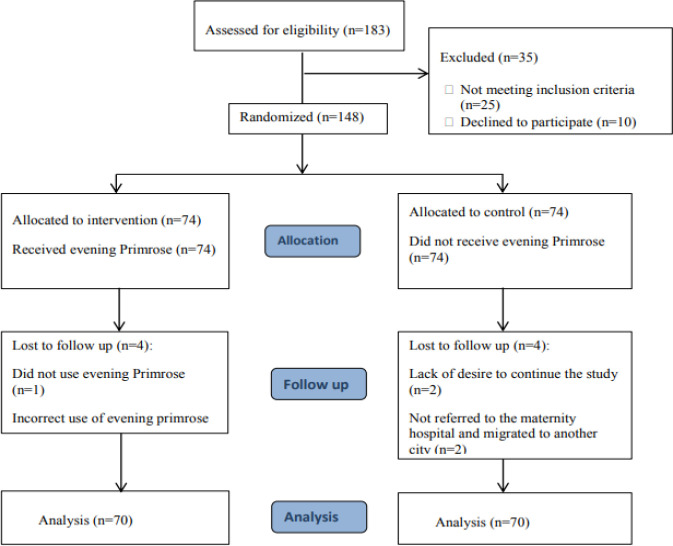
The study CONSORT chart

During the study, 183 subjects were investigated for inclusion into the research, and eventually 74 were randomly assigned to each of the intervention and control group. In each group, 4 subjects were excluded, and analysis was performed for 70 subjects in each group (Figure 1). 

Demographic findings indicated that the mean age in the intervention group was 29.46±6.83 years and in the control group it was 30.43±6.82 years, and the two groups did not show a significant difference in this regard (p=0.401). The two groups were well matched with each other in terms of other personal characteristics (p>0.05) (Table 1).

In five subjects of the evening primrose group, before receiving misoprostol, the abortion process had been started and they did not receive misoprostol, but in the control group all subjects received misoprostol (p<0.05).

According to the results, there was no significant difference between the two groups regarding vital signs or vaginal bleeding before use of misoprostol (p>0.05). Concerning complete abortion, dilatation, and cervix consistency, in the examination before embedding misoprostol, the time between the first dose of misoprostol and discharging the uterus contents, the level of vaginal bleeding during hospitalization, and dose of misoprostol required showed significant differences between the two groups (p<0.05). Regarding duration of hospitalization, curettage, or side effects such as nausea, diarrhea, headache, abdominal cramps, or fever, there was no significant difference between the two groups. The mean pain score was lower in the intervention group compared to the control, though this difference was not significant (p>0.05) (Table 2).

In the intervention group, four subjects had no tissue discharge in response to the medical treatment. This number was 13 in the control group, and there was a significant difference between the two groups in this regard (p<0.05).

**Table 1:** Comparison of the personal characteristics of the intervention (Evening Primrose and misoprostol) and control (misoprostol alone) groups

**Trait**		**Intervention (Evening Primrose and misoprostol)**	**Control (misoprostol alone)**	**p-value**
**BMI (kg/m** ^2^ **)**		Mean±SD 25.78±4.43	Mean±SD 25.71±3.92	0.921^*^
**Gestational ** **age as measured by LMP (weeks)**		11.36±3.99	11±3.66	0.593^*^
**Gestational** ** age ** **as measured by ** **sonography (weeks)**		10.47±9.02	9.53±3.91	0.424^*^
**No of pregnancies**	Primigravida	25 (35.7%)	16 (22.9%)	0.095^**^
	Multigravida	45 (64.3%)	54 (77.1%)	
**No of abortions**	Without history of abortion	50 (71.4%)	47 (67.1%)	0.653^**^
	History of one previous abortion	17 (24.3%)	18 (25.7%)	
	History of two abortions or more	3 (4.3%)	5 (7.1%)	
**History of curettage**	Yes	9 (12.9%)	17 (24.3%)	0.08^**^
	No	61 (87.1%)	53 (75.7%)	
**Type of delivery**	No history of delivery	32 (45.7%)	20 (28.6%)	0.098^**^
	Natural delivery	20 (28.6%)	29 (41.4%)	
	C-section	18 (25.7%)	21 (30%)	

*Independent t-test, **Chi-square test, ***Fisher Exact test. ^1^Body Mass Index. ^2^Last Menstrual Period

**Table 2:** Comparing the studied variables between the intervention (Evening Primrose and misoprostol) and control (misoprostol alone) groups

**Group**		**Intervention (Evening Primrose and misoprostol)**	**Control (misoprostol alone)**	**p-value**
**Misoprostol dose (**μ**g)**		Mean±SD 987.86±559.40	Mean±SD 1275.71±582.46	0.003^*^
**Duration of hospitalization (days)**		2.03±0.24	2.04±0.20	0.704^*^
**The time taken from the first misoprostol** ** dose until fetal ejection (hour:minute)**		8:05±4:33	13:09±8:36	0.000^*^
**Pain score (VAS)**		6.69±2.76	7.18±2.89	0.305^*^
**Vaginal bleeding at the entry**	Without bleeding	46 (65.7%)	51 (72.9%)	0.637^**^
	Spotting	20 (28.6%)	17 (24.3%)	
	Mild	3 (4.3%)	2 (2.9%)	
	Moderate	1 (1.4%)	0 (0%)	
	Sever	0 (0%)	0 (0%)	
**Cervix dilatation**	Open	19 (27.1%)	6 (8.6%)	0.004^**^
	Close	51 (72.9%)	64 (91.4%)	
**Cervix consistency**	Soft	39 (55.7%)	19 (27.1%)	0.001^**^
	Hard	31 (44.3%)	51 (72.9%)	
**Administering misoprostol**	Yes	65 (92.9%)	70 (100%)	0.023^***^
	No	5 (7.1%)	0 (0%)	
**Amount of bleeding during hospitalization**	Mild	63 (90%)	52 (74.3%)	0.043^**^
	Moderate	6 (8.6%)	17 (24.3%)	
	Severe	1 (1.4%)	1 (1.4%)	
**Response to medical treatment**	Yes	66 (94.3%)	53 (75.7%)	0.002^**^
	No	4 (5.7%)	17 (24.3%)	
**Type of abortion**	Complete abortion	8 (11.4%)	1 (1.4%)	0.016^***^
	Incomplete abortion	62 (88.6%)	69 (98.6%)	
**Curettage**	Yes	62 (88.6%)	60 (85.7%)	0.614^**^
	**No**	8 (11.4%)	10 (14.3%)	
**Nausea**	Yes	17(24.3%)	15 (21.4%)	0.687^**^
	**No**	53 (75.7%)	55(78.6%)	
**Headache**	Yes	5(7.1%)	6(8.6%)	0.753^**^
	**No**	65 (92.9%)	64(91.4%)	
**Abdominal cramps**	Yes	8(11.4%)	14 (20%)	0.164^**^
	**No**	62 (88.6%)	56 (80%)	
**Diarrhea**	Yes	19 (27.1%)	20 (28.6%)	0.85^**^
	**No**	51 (72.9%)	50 (71.4%)	
**Fever**	Yes	0 (0%)	1 (1.4%)	0.31^**^
	**No**	70 (100%)	69 (98.6%)	

*Independent t-test, **Chi-square test, ***Fisher Exact test. Visual Analogue Scale

## Discussion

 This study explored the effect of misoprostol with evening primrose and misoprostol alone in treating missed abortion. According to the results, the hybrid treatment had a greater efficacy in treating missed abortion compared to treatment with misoprostol alone. In five subjects (7.1%) of the women in intervention group, upon receiving evening primrose alone, the abortion process was initiated and they did not receive misoprostol. This was unexpected for the researchers since this study was performed presuming that evening primrose could probably boost the effect of misoprostol while it would not be the initiator of abortion. Since its 2000 mg dose could induce abortion in these five people, it seems that using higher doses of evening primrose, percentage of abortion with it and without misoprostol could be increased. In a study by Nouri et al. (2021) ▶, evening primrose was also more effective than misoprostol in cervix dilatation before surgery and the researchers proposed it as a suitable alternative to misoprostol in these cases (Nouri et al., 2021 ▶).

Results showed that the intervention and control groups were significantly different in terms of rate of complete abortion, which was higher in the evening primrose plus misoprostol group compared to the misoprostol alone group. 

The efficacy of the combined treatment of mifepristone and misoprostol has been shown in various studies (Chu et al., 2020 ▶; Warden et al., 2019 ▶). However, considering the higher price of mifepristone compared to evening primrose and its unavailability in many countries as well as the greater tendency of women for using plant-based compounds (Babazadeh and Keramat, 2011 ▶; Movahed et al., 2010 ▶), it seems that evening primrose could be a suitable alternative. The greater efficacy of misoprostol plus letrozole combined treatment has also been shown in some studies (Behroozi-Lak et al., 2018 ▶; Allameh et al., 2020 ▶; Abbasalizadeh et al., 2018 ▶), but the necessity of further studies has also been recommended in this regard (Nash et al., 2018 ▶). In spite of the significant difference between the two groups in terms of complete abortion, the curettage did not differ significantly between them. Possibly this could reflect the over-cautiousness of less experienced gynecologists to ensure completion of abortion before hospital discharge rather than the real need for surgical treatment.

According to the results, dilatation and cervix consistency was significantly different between the evening primrose and control groups. The impact of evening primrose flower on cervix preparation has been confirmed in several studies (Najafi et al., 2019 ▶; Vahdat et al., 2015 ▶; Tanchoco and Aguilar, 2015 ▶), and it was shown to be more effective than misoprostol in some studies (Mirzadeh et al., 2020 ▶). Inconsistent results are related mostly to its oral administration (Jahdi et al., 2016 ▶; Moradi et al., 2021 ▶).

The mean dose of misoprostol used in the present study had significant differences between the intervention and control groups. In another studies also the dose of misoprostol required was lower when given in combination with mifepristone and castor oil compared to misoprostol alone (Chu et al., 2020 ▶; Jamilian and Heydari, 2015 ▶). In a study by Naghshineh et al. (2015) ▶, the administered dose of misoprostol plus letrozole was lower compared to the misoprostol alone, but the difference was not significant (Naghshineh et al., 2015 ▶). With reducing the misoprostol dose, its complication would also diminish while the patient satisfaction will increase (Chen and Creinin, 2007 ▶; Zhang et al., 2005 ▶). The results also indicated fewer complications in the evening primrose group, though the difference between the two groups was not significant.

According to the results, there was a significant difference between the two groups regarding the time interval between administering the first dose of misoprostol until fetal ejection. This is in line with the shorter intervention in the study by Ohannessian et al. (2016) ▶ in misoprostol and mifepristone group compared to treatment with each of them alone for cervix preparation for abortion surgical treatment (Ohannessian et al., 2016 ▶). However, it is incongruent with the findings of Allameh et al. to compared effect of misoprostol alone with misoprostol plus letrozole in treating missed abortion (Allameh et al., 2020 ▶). It can be stated that the results of the present research are more consistent with the study findings of the effect of combined treatment with mifepristone and misoprostol rather than the combined treatment of letrozole plus misoprostol. It seems that combined treatment with evening primrose and misoprostol is preferred over letrozole and misoprostol, though further studies are still required.

In the present study, in spite of the lower pain in the intervention group, the difference between the two groups was not significant. Also, in a study by Shahali et al. (2018) ▶, there was no significant difference between intervention and control groups regarding pain intensity (Shahali et al., 2018 ▶). The pain during speculum insertion and injection for hysterosalpingography was lower in women who received evening primrose in a study by Shahnamnia et al. (2019) ▶ (Shahnamnia et al., 2019 ▶), which is possibly due to the differences in the doses of evening primrose and pain quality between the two studies.

There was no significant difference between the groups of women regarding bleeding before using misoprostol, but bleeding was lower in the hybrid treatment group during hospitalization and when discharging the uterus contents. Considering the importance of midwifery bleeding and its role in maternal mortality, this finding is notable. Bleeding during abortion surgical treatment in a study by Ohannessian et al. (2016) ▶ was also lower in the misoprostol plus mifepristone group compared to the misoprostol plus mifepristone alone (Ohannessian et al., 2016 ▶). In a study by Shahali et al. (2018) ▶ and Kalati et al. (2016) ▶ , although bleeding was lower in the evening primrose group, this difference was not statistically significant (Shahali et al., 2018 ▶; Kalati et al., 2016 ▶).

Use of 2000 mg vaginal evening primrose the night before induction of abortion plus vaginal misoprostol in women suffering from missed abortion had a greater efficacy compared to vaginal misoprostol alone. This could cause higher cases of complete abortion, lower dose of misoprostol required, lower duration of discharging the pregnancy products and less bleeding without increasing the side effects.

Overall, since no study was found regarding the effect of evening primrose plus misoprostol in treating missed abortion, further research is recommended in this regard.

## Conflicts of interest

The authors have declared that there is no conflict of interest.
